# Supplementary material for: Evaluating the Molecular Properties and Function of ANKHD1, and Its Role in Cancer
Source: Int J Mol Sci. 2023 Aug 16;24(16):12834. doi: 10.3390/ijms241612834 (PMC10454556; doi:10.3390/ijms241612834)
Supplement: Supplementary file 1 [file ijms-24-12834-s001.zip › ijms-2529071-supplementary.pdf]

## Supplementary Figure S1

```
Isoform_1    0   MLTDSGGGGTSFEEDLDSVAPRSAPAGASEPPPPGGVGLGIRTVRLFGEAGPASGVGSSGGGGSGSGTGGGDAALDFKLAAAVLRTGGGGGASGSDEDEV   100
Isoform_2    0   MLTDSGGGGTSFEEDLDSVAPRSAPAGASEPPPPGGVGLGIRTVRLFGEAGPASGVGSSGGGGSGSGTGGGDAALDFKLAAAVLRTGGGGGASGSDEDEV   100
Isoform_3    0   MLTDSGGGGTSFEEDLDSVAPRSAPAGASEPPPPGGVGLGIRTVRLFGEAGPASGVGSSGGGGSGSGTGGGDAALDFKLAAAVLRTGGGGGASGSDEDEV   100
Isoform_4    0   MLTDSGGGGTSFEEDLDSVAPRSAPAGASEPPPPGGVGLGIRTVRLFGEAGPASGVGSSGGGGSGSGTGGGDAALDFKLAAAVLRTGGGGGASGSDEDEV   100
Isoform_5    0   MLTDSGGGGTSFEEDLDSVAPRSAPAGASEPPPPGGVGLGIRTVRLFGEAGPASGVGSSGGGGSGSGTGGGDAALDFKLAAAVLRTGGGGGASGSDEDEV   100

Isoform_1  101   SEVESFILDQEDLDNPVLKTTSEIFLSSTAEGADLRTVDPETQARLEALLEAAGIGKLSTADGKAFADPEVLRRLTSSVSCALDEAAAALTRMKAENSHN   200
Isoform_2  101   SEVESFILDQEDLDNPVLKTTSEIFLSSTAEGADLRTVDPETQARLEALLEA-----------AAFADPEVLRRLTSSVSCALDEAAAALTRMKAENSHN   189
Isoform_3  101   SEVESFILDQEDLDNPVLKTTSEIFLSSTAEGADLRTVDPETQARLEALLEAAGIGKLSTADGKAFADPEVLRRLTSSVSCALDEAAAALTRMKAENSHN   200
Isoform_4  101   SEVESFILDQEDLDNPVLKTTSEIFLSSTAEGADLRTVDPETQARLEALLEAAGIGKLSTADGKAFADPEVLRRLTSSVSCALDEAAAALTRMKAENSHN   200
Isoform_5  101   SEVESFILDQEDLDNPVLKTTSEIFLSSTAEGADLRTVDPETQARLEALLEAAGIGKLSTADGKAFADPEVLRRLTSSVSCALDEAAAALTRMKAENSHN   200

Isoform_1  201   AGQVDTRSLAEACSDGDVNAVRKLLDEGRSVNEHTEEGESLLCLACSAGYYELAQVLLAMHANVEDRGNKGDITPLMAASSGGYLDIVKLLLLHDADVNS   300
Isoform_2  190   AGQVDTRSLAEACSDGDVNAVRKLLDEGRSVNEHTEEGESLLCLACSAGYYELAQVLLAMHANVEDRGNKGDITPLMAASSGGYLDIVKLLLLHDADVNS   289
Isoform_3  201   AGQVDTRSLAEACSDGDVNAVRKLLDEGRSVNEHTEEGESLLCLACSAGYYELAQVLLAMHANVEDRGNKGDITPLMAASSGGYLDIVKLLLLHDADVNS   300
Isoform_4  201   AGQVDTRSLAEACSDGDVNAVRKLLDEGRSVNEHTEEGESLLCLACSAGYYELAQVLLAMHANVEDRGNKGDITPLMAASSGGYLDIVKLLLLHDADVNS   300
Isoform_5  201   AGQVDTRSLAEACSDGDVNAVRKLLDEGRSVNEHTEEGESLLCLACSAGYYELAQVLLAMHANVEDRGNKGDITPLMAASSGGYLDIVKLLLLHDADVNS   300

Isoform_1  301   QSATGNTALTYACAGGFVDIVKVLLNEGANIEDHNENGHTPLMEAASAGHVEVARVLLDHGAGINTHSNEFKESALTLACYKGHLDMVRFLLEAGADQEH   400
Isoform_2  290   QSATGNTALTYACAGGFVDIVKVLLNEGANIEDHNENGHTPLMEAASAGHVEVARVLLDHGAGINTHSNEFKESALTLACYKGHLDMVRFLLEAGADQEH   389
Isoform_3  301   QSATGNTALTYACAGGFVDIVKVLLNEGANIEDHNENGHTPLMEAASAGHVEVARVLLDHGAGINTHSNEFKESALTLACYKGHLDMVRFLLEAGADQEH   400
Isoform_4  301   QSATGNTALTYACAGGFVDIVKVLLNEGANIEDHNENGHTPLMEAASAGHVEVARVLLDHGAGINTHSNEFKESALTLACYKGHLDMVRFLLEAGADQEH   400
Isoform_5  301   QSATGNTALTYACAGGFVDIVKVLLNEGANIEDHNENGHTPLMEAASAGHVEVARVLLDHGAGINTHSNEFKESALTLACYKGHLDMVRFLLEAGADQEH   400

Isoform_1  401   KTDEMHTALMEACMDGHVEVARLLLDSGAQVNMPADSFESPLTLAACGGHVELAALLIERGANLEEVNDEGYTPLMEAAREGHEEMVALLLAQGANINAQ   500
Isoform_2  390   KTDEMHTALMEACMDGHVEVARLLLDSGAQVNMPADSFESPLTLAACGGHVELAALLIERGANLEEVNDEGYTPLMEAAREGHEEMVALLLAQGANINAQ   489
Isoform_3  401   KTDEMHTALMEACMDGHVEVARLLLDSGAQVNMPADSFESPLTLAACGGHVELAALLIERGANLEEVNDEGYTPLMEAAREGHEEMVALLLAQGANINAQ   500
Isoform_4  401   KTDEMHTALMEACMDGHVEVARLLLDSGAQVNMPADSFESPLTLAACGGHVELAALLIERGANLEEVNDEGYTPLMEAAREGHEEMVALLLAQGANINAQ   500
Isoform_5  401   KTDEMHTALMEACMDGHVEVARLLLDSGAQVNMPADSFESPLTLAACGGHVELAALLIERGANLEEVNDEGYTPLMEAAREGHEEMVALLLAQGANINAQ   500

Isoform_1  501   TEETQETALTLACCGGFSEVADFLIKAGADIELGCSTPLMEASQEGHLELVKYLLASGANVHATTATGDTALTYACENGHTDVADVLLQAGADLEHESEG   600
Isoform_2  501   TEETQETALTLACCGGFSEVADFLIKAGADIELGCSTPLMEASQEGHLELVKYLLASGANVHATTATGDTALTYACENGHTDVADVLLQAGADLDKQEDM   600
Isoform_3  490   TEETQETALTLACCGGFSEVADFLIKAGADIELGCSTPLMEASQEGHLELVKYLLASGANVHATTATGDTALTYACENGHTDVADVLLQAGADLDKQEDM   589
Isoform_4  501   TEETQETALTLACCGGFSEVADFLIKAGADIELGCSTPLMEASQEGHLELVKYLLASGQAGGHEDYFGGHRSGQASGEGGL-------------------   581
Isoform_5  501   TEETQETALTLACCGGFSEVADFLIKAGADIELGCSTPLMEASQEGHLELVKYLLASGANVHATTATGDTALTYACENGHTDVADVLLQAGADLEHESEG   600

Isoform_1  601   GRTPLMKAARAGHLCTVQFLISKGANVNRATANNDHTVVSLACAGGHLAVVELLLAHGADPTHRLKDGSTMLIEAAKGGHTNVVSYLLDYPNNVLSVPTT   700
Isoform_2  590   KT-ILEGIDPAKHQVRVAFDACKLLRKE------------------------------------------------------------------------   616
Isoform_3  601   KT-ILEGIDPAKHQVRVAFDACKLLRKE------------------------------------------------------------------------   627
Isoform_5  601   GRTPLMKAARAGHLCTVQFLISKGANVNRATANNDHTVVSLACAGGHLAVVELLLAHGADPTHRLKDGSTMLIEAAKGGHTNVVSYLLDYPNNVLSVPTT   700

Isoform_1  701   DVSQLPPPSQDQSQVPRVPTHTLAMVVPPQEPDRTSQENSPALLGVQKGTSKQKSSSLQVADQDLLPSFHPYQPLECIVEETEGKLNELGQRISAIEKAQ   800
Isoform_5  701   DVSQLPPPSQDQSQVPRVPTHTLAMVVPPQEPDRTSQENSPALLGVQKGTSKQKSSSLQVADQDLLPSFHPYQPLECIVEETEGKLNELGQRISAIEKAQ   800
```
